# Efferent and Afferent Connections of Neuropeptide Y Neurons in the Nucleus Accumbens of Mice

**DOI:** 10.3389/fnana.2021.741868

**Published:** 2021-09-10

**Authors:** Shunji Yamada, Nienke van Kooten, Takuma Mori, Katsutoshi Taguchi, Atsushi Tsujimura, Masaki Tanaka

**Affiliations:** ^1^Department of Anatomy and Neurobiology, Graduate School of Medical, Kyoto Prefectural University of Medicine, Kyoto, Japan; ^2^Department of Molecular and Cellular Physiology Shinshu University, School of Medicine, Matsumoto, Japan; ^3^Department of Basic Geriatrics, Graduate School of Medical Science, Kyoto Prefectural University of Medicine, Kyoto, Japan

**Keywords:** Neuropeptide Y, nucleus accumbens, lateral hypothalamus, adeno-associated virus, recombinant rabies virus, Cre-LoxP

## Abstract

Neuropeptide Y (NPY) is a neural peptide distributed widely in the brain and has various functions in each region. We previously reported that NPY neurons in the nucleus accumbens (NAc) are involved in the regulation of anxiety behavior. Anterograde and retrograde tracing studies suggest that neurons in the NAc project to several areas, such as the lateral hypothalamus (LH) and ventral pallidum (VP), and receive afferent projections from the cortex, thalamus, and amygdala. However, the neural connections between accumbal NPY neurons and other brain areas in mice remain unclear. In this study, we sought to clarify these anatomical connections of NPY neurons in the NAc by investigating their neural outputs and inputs. To selectively map NPY neuronal efferents from the NAc, we injected Cre-dependent adeno-associated viruses (AAVs) into the NAc of NPY-Cre mice. This revealed that NAc NPY neurons exclusively projected to the LH. We confirmed this by injecting cholera toxin b subunit (CTb), a retrograde tracer, into the LH and found that approximately 7–10% of NPY neurons in the NAc were double-labeled for mCherry and CTb. Moreover, retrograde tracing using recombinant rabies virus (rRABV) also identified NAc NPY projections to the LH. Finally, we investigated monosynaptic input to the NPY neurons in the NAc using rRABV. We found that NPY neurons in the NAc received direct synaptic connections from the midline thalamic nuclei and posterior basomedial amygdala. These findings provide new insight into the neural networks of accumbal NPY neurons and should assist in elucidating their functional roles.

## Introduction

Neuropeptide Y (NPY) is a neural peptide distributed widely in the brain and is well known for its role in appetite, maintenance of energy homeostasis, and anxiety. For example, NPY-expressing neurons in the arcuate nucleus of the hypothalamus (ARC) co-express agouti-related peptide (AgRP), and their activation causes an increase in food intake (Clark et al., 1984; Aponte et al., 2011). Microinjection of NPY into the amygdala has an anxiolytic effect in rats (Heilig et al., 1993). Recently, we performed time- and region-specific disruption and chemogenetic activation of NPY neurons in the NAc using NPY-Cre mice, which revealed that NAc NPY neurons have a role in reducing anxiety (Yamada et al., 2020).

There are many NPY-positive (Chronwall et al., 1985; Yamada et al., 2020) and NPY mRNA-expressing cells in the NAc (Morris, 1989). Kawaguchi et al. (1995) showed that a subset of GABA and somatostatin double-positive interneurons also express NPY in the neostriatum. These results suggest that NPY neurons in the NAc are interneurons. We previously demonstrated that overexpression of NPY in the NAc ameliorates behavioral despair in mice (Aoki et al., 2016). Because NPY or NPY receptor type 1 (Y1-R) agonist injection into the NAc did not affect behavioral despair, we proposed that some NPY neurons in the NAc were projection neurons (Aoki et al., 2016).

Previous reports using an anterograde tracer, biotinylated dextran amine (BDA), suggest that neurons in the NAc project to various regions, such as the ventral pallidum (VP), the lateral hypothalamus (LH), the entopeduncular nucleus, and substantia nigra pars compacta, in rats (Heimer et al., 1991; Usuda et al., 1998). The NAc receives afferent projections from the medial prefrontal cortex (mPFC), the amygdala, the hippocampus, and midline thalamic nuclei in rats (Brog et al., 1993). Moreover, a recent report using retrograde adeno-associated virus (AAV-retro) showed that the NAc receives projections from various cortical areas such as the mPFC, thalamus, amygdala, and CA1 region in mice (Itoga et al., 2019).

In this study, to provide more anatomical information on the connections of NPY neurons in the NAc, we investigated the projections of these cells using NPY-Cre mice and viral genetic mapping approaches. Moreover, we investigated monosynaptic input to the NPY neurons in the NAc using recombinant rabies virus (rRABV).

## Materials and Methods

### Animals

To produce NPY-Cre mice, B6.FVB(Cg)-Tg(NPY-cre)RH26Gsat/Mmucd (037423-UCD, NPY-Cre) sperm were purchased from Mutant Mouse Resource & Research Centers (Auckland, CA, USA). We entrusted the generation of NPY-Cre mice from the sperm to the RIKEN BioResource Research Center (Ibaraki, Japan). The mice were kept under a 12-h light/dark cycle (lights on at 8 a.m.). Standard food pellets and water were provided *ad libitum*. All animal experiments, such as production, maintenance protocols, and behavioral studies, were reviewed and approved by the Animal Care and Use Committee of the Kyoto Prefectural University of Medicine (M2019-155&157, M2020-162&165).

### Adeno-Associated Virus Preparation

pAAV-EF1a-DIO-mCherry (Addgene plasmid# 50462; http://n2t.net/addgene:50462; RRID: Addgene_50462) was a gift from Bryan Roth. pAAV-phSyn1(S)-FLEX-tdTomato-T2A-SypEGFP-WPRE was a gift from Hongkui Zeng (Addgene plasmid# 51509; http://n2t.net/addgene:51509; RRID: Addgene_51509) (Oh et al., 2014). pAAV-EF1a-FLEX-TVA-mCherry was a gift from Naoshige Uchida (Addgene plasmid# 38044; http://n2t.net/addgene:38044; RRID: Addgene_38044) (Watabe-Uchida et al., 2012). pAAV-FLEX-SADcvsG was generated (Mori and Morimoto, 2014). For recombinant AAV (rAAV) production, the vectors were cotransfected into HEK293 cells with pAAV-DJ (Cell Biolabs, San Diego, CA, USA) and pHelper (Takara Bio Inc., Shiga, Japan) using AAVpro Helper-Free system (Takara Bio Inc.). rAAV particles were extracted and purified using the AAVpro Purification Kit (Takara Bio Inc.), according to the instructions of the manufacturer. rAAV titers were determined by quantitative PCR using the AAVpro Titration Kit (Takara Bio Inc.), and aliquoted rAAVs were stored at −80°C.

### Recombinant Rabies Virus Production

We produced the HEP-Flury (HEP) strain RABV using plasmid encoding glycoprotein-deficient HEP-Flury expressing GFP (pcDNA-HEPdG-GFP), helper plasmids encoding SADcvsG, which is the cytoplasmic domain of SAD glycoprotein replaced by that of CVSG (pCAGGS-SADcvsG), helper plasmids encoding HEP nucleoprotein (pcDNA-HEPN), phosphoprotein (pcDNA-HEPP), and polymerase (pcDNA-HEPL) as described in our previous study (Mori and Morimoto, 2014). About 1.0 × 10^4^ BHK-T7/9 cells, expressing T7 RNA polymerase, were seeded into each well of a 100-mm dish in the medium containing 2% fetal bovine serum (FBS). Cells were transfected with plasmid encoding pcDNA-HEP-ΔG-GFP (2 μg), pcDNA-HEPN (1 μg), pcDNA-HEPP (0.5 μg), pcDNA-HEPL (0.25 μg), and pCAGGS-SADcvsG (0.5 μg) using TransIT-LT1 (Mirus Bio LLC, Madison, WI, USA), as described by the manufacturer. The plates were incubated for 2 days at 32°C in an atmosphere containing 5% CO_2_, and then, the supernatant was collected as HEPdG-GFP. To produce EnvA-enveloped HEPdG-GFP, HEPdG-GFP was added to wells of 80% confluent BHK-T7/9 cells on 100-mm plates and then incubated for 2 days at 32°C in an atmosphere containing 5% CO_2_. Thereafter, the supernatant was removed, and the cells were washed with PBS (5×) to eliminate the remaining HEPdG-GFP and incubated as before. The next day, the HEPdG-GFP-infected cells were transfected with pCAGGS-EnvA and incubated for 4 days. The supernatant containing EnvA-HEPdG-GFP was concentrated by ultracentrifugation, and aliquots of EnvA-HEPdG-GFP were stored at −80°C.

### Stereotaxic Virus Injections

Mice were anesthetized with a midazolam/medetomidine/butorphanol cocktail (4, 0.3, and 5 mg/kg, respectively) intraperitoneally and placed on a stereotaxic apparatus (Narishige, Tokyo, Japan). AAVs were unilaterally microinjected (0.2–1 μl/site) into the NAc (AP, +1.2 mm from the bregma; ML, +0.8 mm from the midline; and DV, 4.3 mm below the skull surface) using a 30-gauge Hamilton syringe needle (Hamilton, Reno, NV, USA) at a rate of 0.1 μl/min. The needle was kept in place for 5–10 min after each injection before the needle was slowly removed. Some mice that received a second brain injection were housed individually, while the others were returned to their group home cage for recovery. Viral injections were verified by fluorescent image and location of cannula tip, and data from mice that were not correctly injected virus and CTb into the target region were excluded.

### Visualization and Anterograde Labeling of NPY Neurons in the NAc

AAV(dj)-FLEX-mCherry (0.8 × 10^7^ viral genome particles/ml) or AAV(dj)-FLEX-tdTomato-T2A-SypEGFP (2.7 × 10^11^ viral genome particles/ml) of 0.5 μl was injected into the NAc (*n* = 3 each). mCherry, tdTomato, and synaptophysin-EGFP (sypEGFP) expressions were allowed to develop for more than 2 weeks before the mice were deeply anesthetized and perfused.

### Retrograde Tracing With CTb

To label NAc NPY neurons, 0.5 μl of AAV(dj)-FLEX-mCherry was injected into the NAc. Two weeks later, 0.5 μl of CTb (10 μg/μl; C-7771, Sigma-Aldrich, Steinheim, Germany), a retrograde tracer, was injected into the LH (AP, −1.2 mm from the bregma; ML, +0.9 mm from the midline; and DV, 4.3 mm below the skull surface) at a rate of 0.1 μl/min. CTb was allowed to undergo retrograde transport from the LH to the NAc for 6 days before the mice were deeply anesthetized and perfused (*n* = 5).

### Retrograde Tracing With rRABV

AAV(dj)-FLEX-TVA-mCherry (1.5 × 10^11^ viral genome particles/ml) of 0.5 μl was injected into the NAc. Two weeks later, 1 μl of EnvA-HEPdG-GFP was injected into the LH, and the virus was allowed to undergo retrograde transport from the LH to the NAc for 1 week before the mice were deeply anesthetized and perfused (*n* = 4).

### Monosynaptic Retrograde Tracing Using rRABV

To target global input to NPY neurons in the NAc, we performed monosynaptic retrograde tracing (Watabe-Uchida et al., 2012; Krashes et al., 2014). A mixture containing 0.5 μl of AAV(dj)-FLEX-TVA-mCherry and AAV(dj)-FLEX-SADcvsG (2.7 × 10^11^ viral genome particles/ml) was injected into the NAc. Then, 2 weeks later, 1 μl of EnvA-HEPdG-GFP was injected into the same region, and the virus was allowed to undergo retrograde transport for 1 week before the mice were deeply anesthetized and perfused (*n* = 6).

### Immunohistochemistry (IHC)

Mice were anesthetized with pentobarbital (Somnopentyl; Kyoritsu Seiyaku, Tokyo, Japan) intraperitoneally and perfused with physiological saline followed by 4% paraformaldehyde in 0.05 M phosphate buffer. The brain was immediately removed, postfixed with the same fixative overnight at 4°C, and then kept in 30% sucrose in 0.05 M phosphate buffer at 4°C. Serial coronal sections (40 μm) were obtained using a cryostat (CM 3050 S; Leica, Wetzlar, Germany).

For visualization and anterograde tracing of NPY neurons in the NAc, we performed mCherry IHC. Every fourth section was sequentially incubated with 0.3% H_2_O_2_ and 0.3% Triton X-100 in PBS for 30 min, and then in PBS containing 2% normal goat serum (NGS) and 0.1% Triton X-100 for 1 h at room temperature (RT). Sections were then incubated with primary rabbit antiserum against mCherry (1:2,000; ab167453, Abcam, Cambridge, UK) for 72 h at 4°C. Immunoreactive neurons were visualized with a streptavidin-biotin kit (Nichirei, Tokyo, Japan), followed by 3,3′-diaminobenzidine (DAB), as described in our previous studies (Takanami et al., 2010; Yamada and Kawata, 2014).

To confirm AAV(dj)-FLEX-tdTomato-T2A-SypEGFP infection, the brain sections were processed for GFP immunofluorescence. Every fourth section was incubated with 2% NGS in PBS for 1 h and then incubated with primary rabbit antiserum against GFP (1:2,000; ab6556, Abcam) for 72 h at 4°C. After washing with PBS, the sections were incubated with Alexa Fluor 488-labeled donkey anti-rabbit IgG (1:1,000; A21206, Thermo Fisher Scientific, MA, USA).

For mCherry and CTb double IHC, every fourth section from the NAc to LH was incubated with 2% normal rabbit serum (NRS) in PBS for 1 h and then incubated with primary goat antiserum against CTb (1:1,000; #703, List Biological Laboratories Inc., CA, USA) and rat antiserum against mCherry (1:2,000; M11217, Thermo Fisher Scientific) for 72 h at 4°C. After washing with PBS, the sections were incubated with Alexa Fluor 488-labeled donkey anti-goat IgG (1:1,000; ab150129, Abcam) and Alexa Fluor 555-labeled donkey anti-rat IgG (1:1,000; ab150154, Abcam).

For mCherry and GFP double IHC, every fourth section was incubated with 2% NGS in PBS for 1 h and then with primary rabbit antiserum against GFP (1:2,000) and rat antiserum against mCherry (1:2,000) for 72 h at 4°C. After washing with PBS, the sections were incubated with Alexa Fluor 488-labeled donkey anti-rabbit IgG (1:1,000) and Alexa Fluor 555-labeled donkey anti-rat IgG (1:1,000).

### Statistical Analysis

Fluorescence images of high magnification were obtained on an LSM510META confocal laser-scanning microscope (Carl Zeiss, Jena, Germany). Fluorescence images of low magnification were observed under a fluorescence microscope (CKX53; Olympus, Tokyo, Japan), and images were captured using a CCD camera (DP 74; Olympus). Fluorescence intensities were manipulated to make clear the injection sites and location of neural nuclei in images of low magnification. The sections reacted with DAB were observed under a light microscope (BX50; Olympus), and images were captured using a CCD camera (DP 21; Olympus). To evaluate the numbers of labeling cells and the ratio, we determined the region of interest (ROI) in the medial part of the NAc. The size of ROI is defined as 0.45 mm × 0.45 mm, and this is the size of the photo frame in our confocal laser-scanning microscope using a 20 × objective lens. This frame is able to cover the medial part of NAc where is the target region of AAV. mCherry-positive, GFP-positive, mCherry and CTb double-positive, and mCherry and GFP double-positive cells were counted in these images. All values are expressed as mean ± SEM.

## Results

### Visualization and Anterograde Tracing of NPY Neurons in the NAc

**Figure 1:**
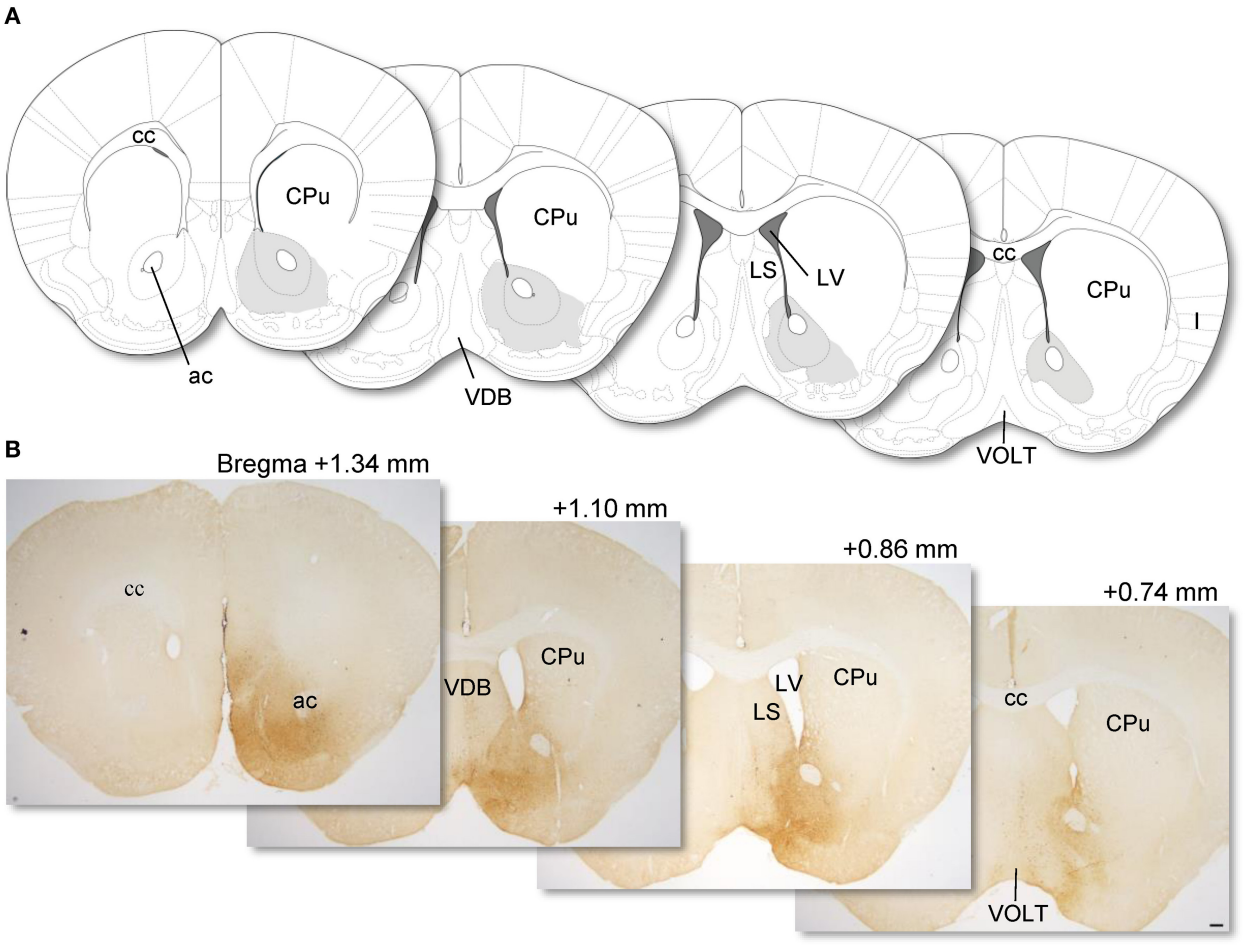
Coronal sections from the mouse brain atlas of Franklin and Paxinos (2007) showing the position of the NAc **(A)**. Representative photomicrographs showing the site of mCherry expression in the NAc (dark brown areas) in AAV-FLEX-mCherry-injected NPY-Cre mice (injection into the NAc) **(B)**. Numerical values on each photomicrograph show distance from bregma (mm). Scale bar, 100 μm. ac, anterior commissure; cc, corpus callosum; CPu, caudate putamen; I, insular cortex; LS, lateral septum; LV, lateral ventricular; VDB, nucleus of the vertical limb of the diagonal band; VOLT, vascular organ of the lamina terminalis.

To visualize NPY neurons in the NAc, we injected AAV(dj)- FLEX-mCherry into the NAc in NPY-Cre mice, and mCherry was visualized by IHC. Previously, we confirmed that our AAV injection into the NAc of NPY-Cre mice caused specific expression of mCherry in accumbal NPY neurons (Yamada et al., 2020). We observed that a 0.2-μl volume of AAV injection at 1.2-mm anterior from the bregma spread through the NAc, with a longitudinal distance from 1.34 mm to 0.74 mm anterior to the bregma in the brain atlas (Franklin and Paxinos, 2007). NPY neurons in the medial part of the NAc were labeled by the injection without distinction of the core and shell of the nucleus (Figure 1). Other than NAc, we found a few mCherry-positive cells in the lateral septum (LS) and nucleus of the vertical limb of the diagonal band (VDB).

**Figure 2:**
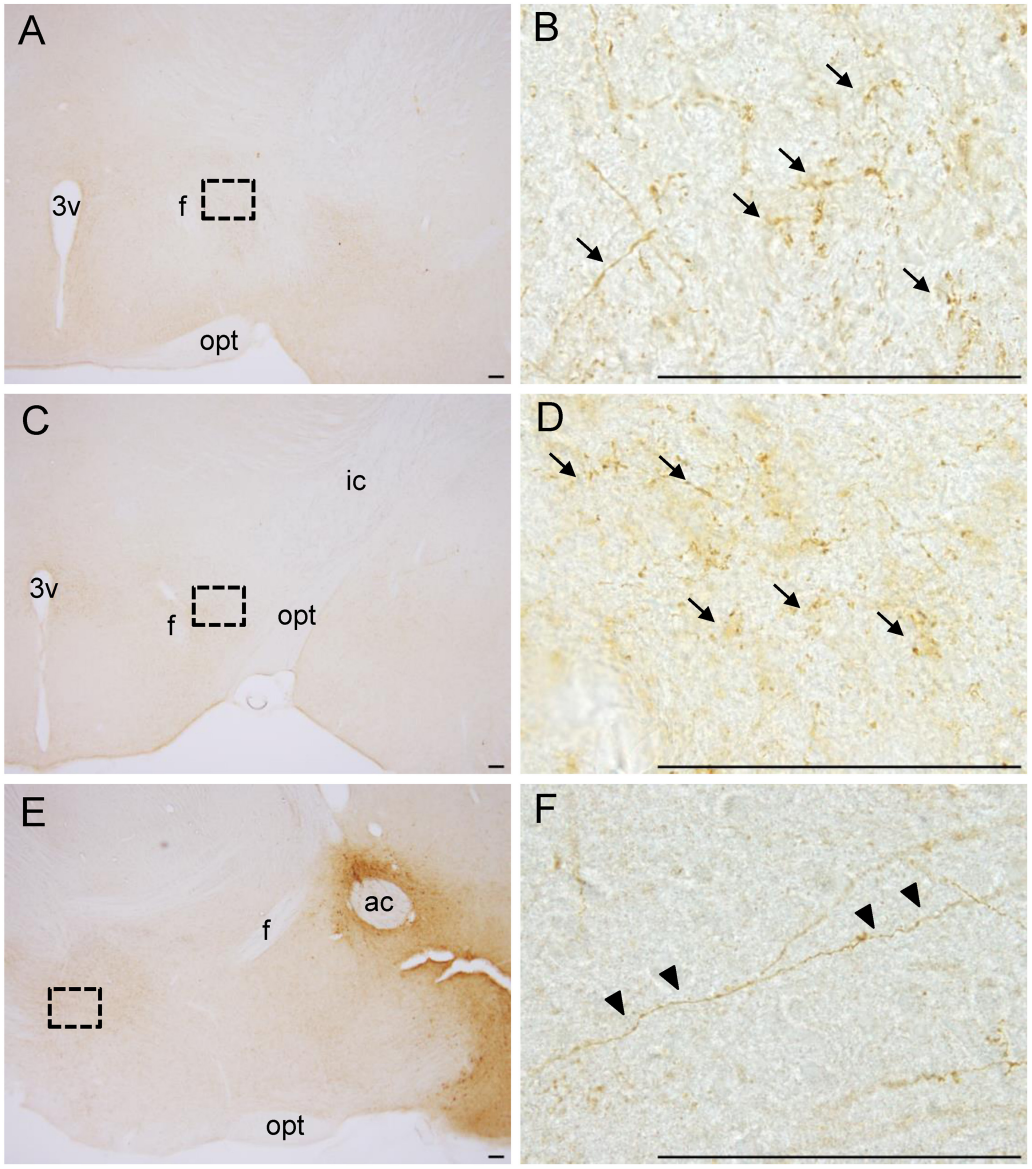
Photographs of dot-like mCherry-expressing fibers (arrows) from the NAc in the anterior part **(A, B)** and posterior part **(C, D)** of the LH in coronal sections. In sagittal sections **(E, F)**, mCherry-expressing fibers from the NAc to the LH are shown (arrowheads). The regions in the black square boxes in **A, C**, and **E** are magnified in **B, D**, and **F**, respectively. Scale bars, 100 μm. ac, anterior commissure; f, fornix; ic, internal capsule; opt, optic tract; 3v, third ventricle.

We analyzed mCherry projections originating from the NAc in whole-brain sections. We found many dot-like mCherry-expressing fibers in the anterior (Figures 2A,B) and posterior (Figures 2C,D) parts of the LH in coronal sections. In sagittal sections, we also found some mCherry-expressing fibers in the LH (Figures 2E,F). In addition, we found a few mCherry-expressing fibers around the LH, such as the VP and extended amygdala. mCherry-positive cell bodies and fibers were dominant in the NAc and LH, respectively, demonstrating that NPY neurons in the NAc project to the LH.

**Figure 3:**
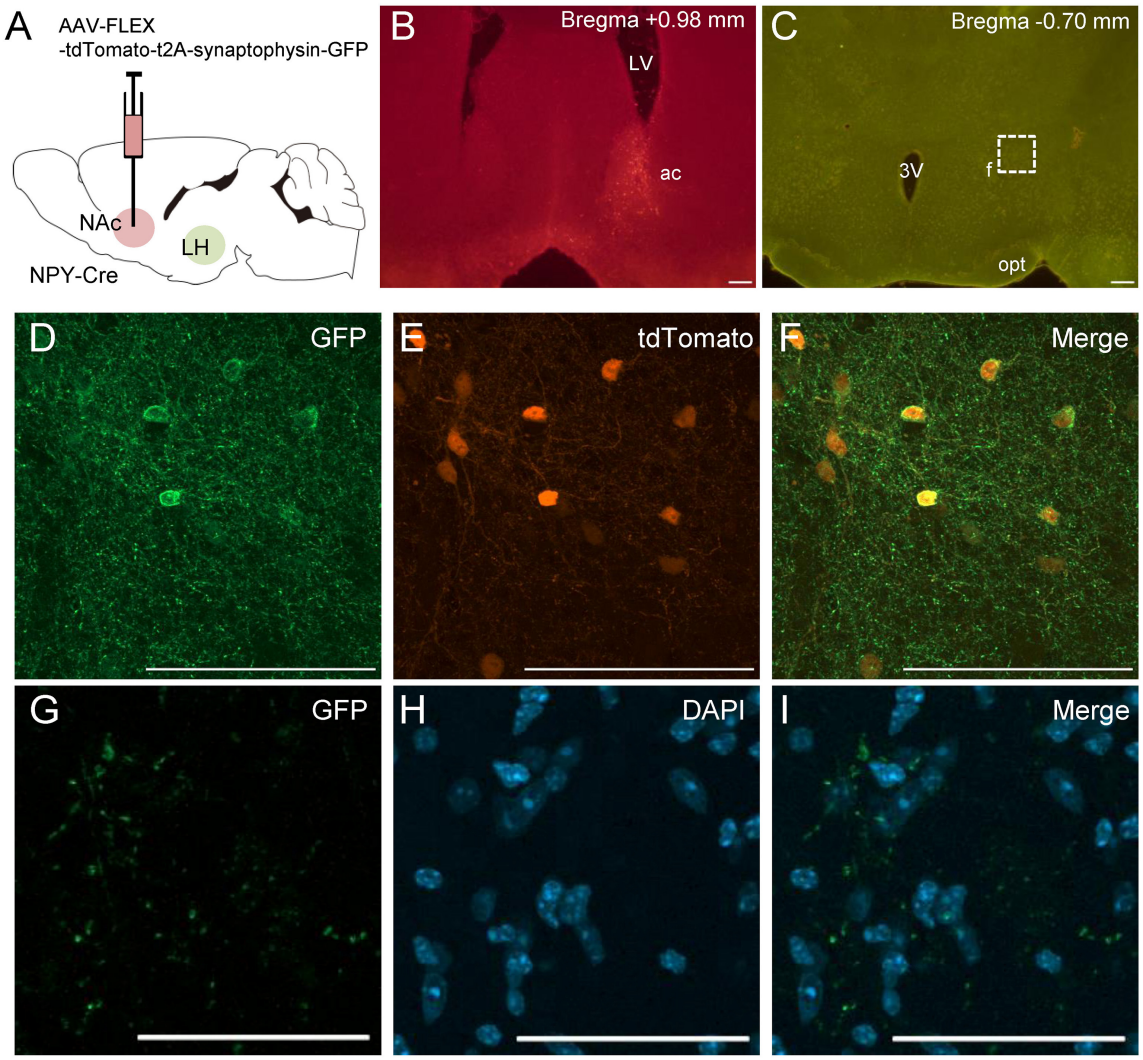
Schematic illustration of AAV(dj)-FLEX-tdTomato-sypEGFP injection into the NAc in NPY-Cre mice **(A)**. Representative photograph of tdTomato-expressing cell bodies at the NAc injection site **(B)**, and of sypEGFP-expressing fibers in the LH **(C)**. Magnified fluorescence image of GFP **(D)**, tdTomato **(E)**, and merged **(F)** in the NAc. The regions in the white square box in C are magnified in **G**–**I**. Scale bars, 100 μm (**B**–**F**) and 50 μm (**G**–**I**). Numerical values in each photomicrographs show distances from bregma (mm). ac, anterior commissure; f, fornix; LV, lateral ventricle; opt, optic tract; 3v, third ventricle.

To confirm the finding of NPY neural connections between the NAc and the LH, we injected other Cre-dependent AAV into the NAc in NPY-Cre mice (Figure 3A). This AAV encoded the synaptic protein synaptophysin fused with EGFP (sypEGFP; AAV-FLEX-tdTomato-sypEGFP) (Oh et al., 2014). Many double-labeled cell bodies for tdTomato and sypEGFP were detected in the NAc around the injection site (Figures 3B,D–F). In these mice, we also observed EGFP-positive presynaptic puncta in the LH (Figures 3C,G–I).

### Retrograde Tracing of NAc NPY Neuronal Projections to the LH Using CTb

**Figure 4:**
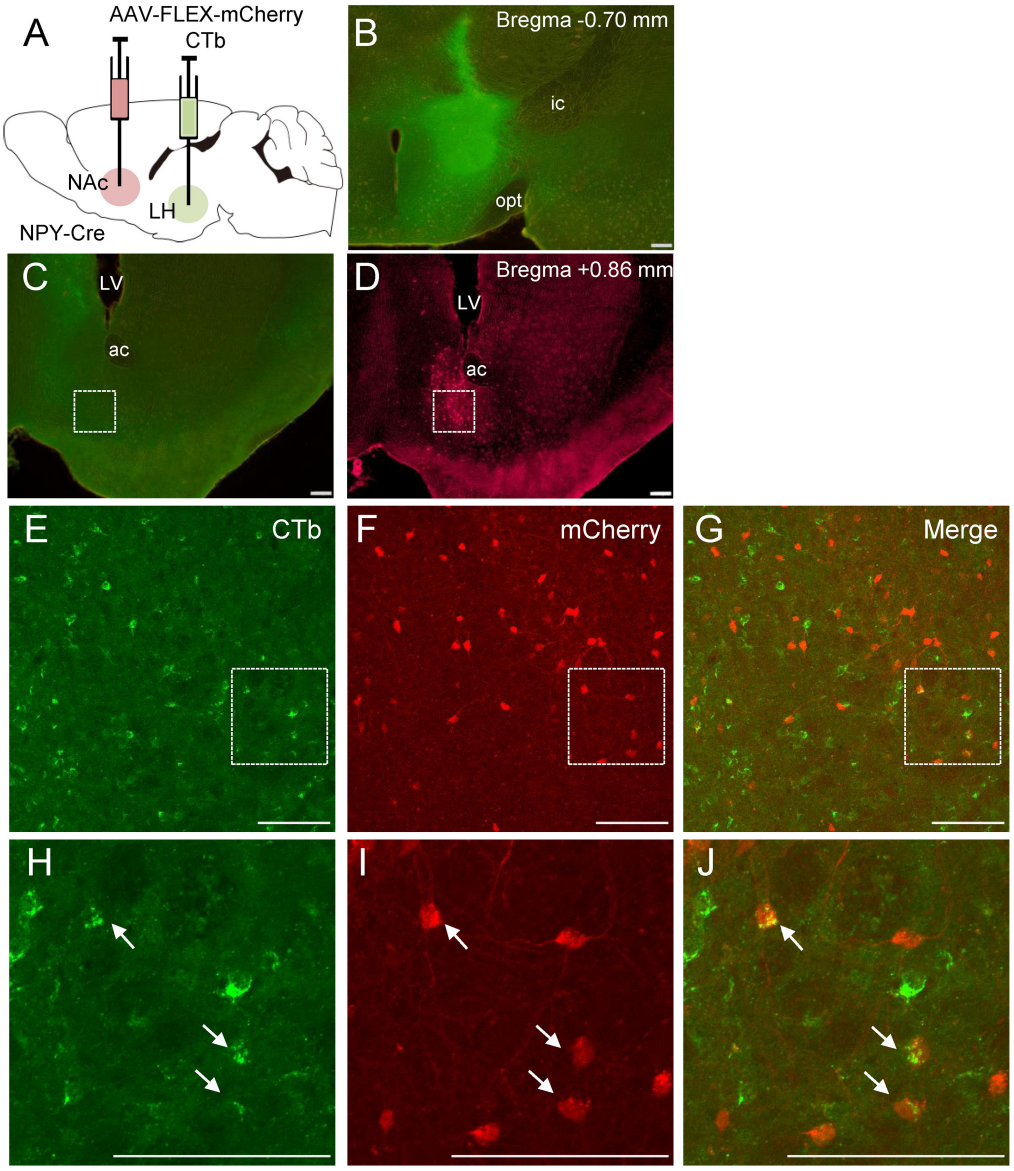
Schematic illustration of AAV(dj)-FLEX-mCherry injection into the NAc and CTb injection into the LH in NPY-Cre mice **(A)**. Representative photograph of the CTb injection site **(B)**. Representative photographs showing the site of CTb **(C)** and mCherry **(D)** expression in the NAc. Magnified fluorescence image of CTb **(E)**, mCherry **(F)**, and merged **(G)** in the white square box in **C** and **D**. The regions in the white square boxes in **E**, **F**, and **G** are more magnified in **H, I**, and **J**, respectively. Arrows show CTb and mCherry double-positive neurons. Scale bars, 100 μm. Numerical values in each photomicrograph show distance from bregma (mm). ac, anterior commissure; f, fornix; ic, internal capsule; LV, lateral ventricle; opt, optic tract.

To further clarify the neuroanatomical connections of NPY neurons between the NAc and LH, we performed retrograde tracing using CTb (*n* = 5). NPY is a neurotransmitter that is released from axon terminals and is usually transported to the pre-synapse after synthesis and modification of the precursor peptide within the endoplasmic reticulum and Golgi apparatus (von Hörsten et al., 2004). To visualize NPY neuron cell bodies, we injected Cre-dependent AAV encoding mCherry into the NAc in NPY-Cre mice 2 weeks before CTb injection into the LH (Figure 4A). Injection of CTb into the LH (Figure 4B) caused the ipsilateral distribution of CTb-positive neurons in the NAc (Figures 4C,E,H). We also found several CTb and mCherry double-positive neurons in the NAc (Figures 4G,J). The numbers of mCherry-positive and CTb and mCherry double-positive neurons in the ROI (0.45 × 0.45 mm) in the NAc were 142.0 ± 27.9 and 10.2 ±2.52, respectively. The percentage of CTb-positive neurons among total mCherry-positive neurons was 7.19 ± 1.16% (Table 1).

**Table 1:** Numbers and percentages of mCherry and CTb-immunoreactive neurons in the NAc after CTb injection into the LH.

**Numbers of mCherry-ir neurons**	**Numbers of CTb and mCherry double-ir neurons**	**Percentage of CTb-ir in mCherry-ir neurons (%)**
142.0 ± 27.9	10.2 ± 2.52	7.19 ± 1.16

*n = 5, 3–5 sections for each mouse. ir, immunoreactive*.

### Retrograde Tracing of NAc NPY Neuronal Projections to the LH Using rRABV With the EnvA-TVA System

**Figure 5:**
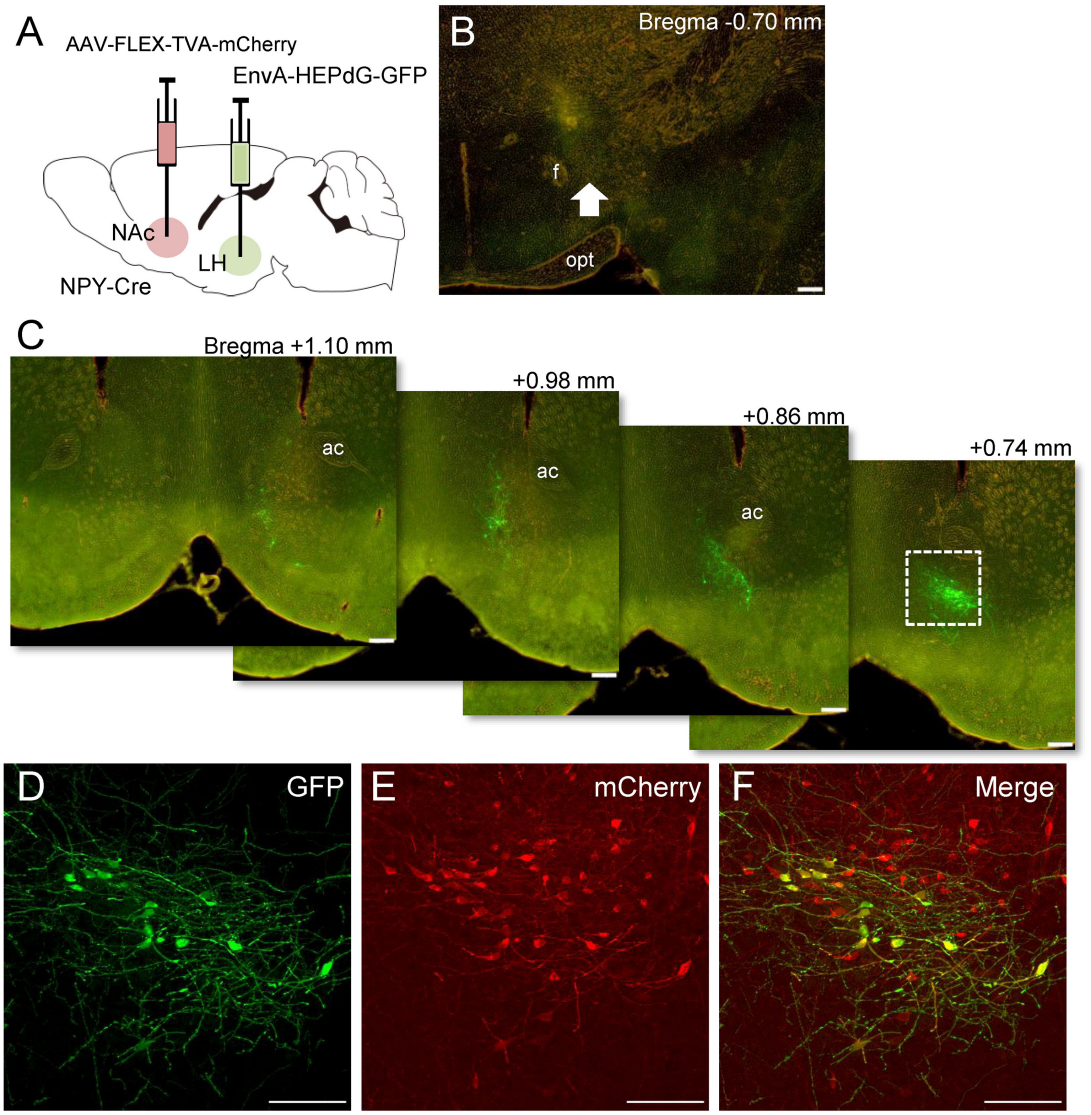
Schematic illustration of AAV(dj)-FLEX-TVA-mCherry injection into the NAc and EnvA-HEPdG-GFP injection into the LH in NPY-Cre mice **(A)**. Representative photograph of the EnvA-HEPdG-GFP injection site **(B)**. The arrow shows the injection site. Representative photographs showing the site of GFP expression through the rostral to caudal regions of the NAc **(C)**. Magnified fluorescence image of GFP **(D)**, mCherry **(E)**, and merged **(F)** in the white square box in C. Scale bars, 100 μm. Numerical values in or on each photomicrographs show distances from bregma (mm). ac, anterior commissure; f, fornix; opt, optic tract.

We investigated whether other retrograde tracing methods would also reveal projections of NAc NPY neurons to the LH (*n* = 4). To this end, we employed Cre-dependent AAV encoding avian sarcoma leukosis virus receptor TVA (AAV-FLEX-TVA-mCherry) and avian sarcoma leukosis virus envelop protein (EnvA)-enveloped RABVdG (EnvA-HEPdG-GFP) (Sun et al., 2019). First, AAV-FLEX-TVA-mCherry was injected into the NAc in NPY-Cre mice to express TVA in NPY neural cell bodies and terminals, and then, EnvA-HEPdG-GFP was injected into the LH (Figures 5A,B). If there are neural terminals containing TVA from accumbal NPY neurons, EnvA-HEPdG-GFP would infect *via* TVA and get retrogradely transported to the NAc. We found that a considerable number of GFP-positive neurons were distributed ipsilaterally through the rostral to caudal regions of the NAc, with caudal dominance (Figure 5C). We also observed that most GFP-positive neurons in the NAc overlapped with mCherry-positive neurons (Figures 5D–F). The numbers of mCherry-positive and GFP and mCherry double-positive neurons in the ROI (0.45 × 0.45 mm) in the NAc were 99.5 ± 12.1 and 10.3 ±3.5, respectively. The percentage of GFP-positive neurons among total mCherry-positive neurons was 9.55 ± 2.15% (Table 2). When AAV-FLEX-TVA-mCherry or EnvA-HEPdG-GFP was injected into the NAc in wild-type mice, there was no mCherry- or GFP-positive neurons in the NAc (data not shown) indicating viral specificity for Cre or TVA expressing cells, respectively.

**Table 2:** Numbers and percentages of mCherry (NPY) and GFP-immunoreactive neurons in the NAc after EnvA-HEPdG-GFP injection into the LH.

**Numbers of mCherry-ir neurons**	**Numbers of GFP and mCherry double-ir neurons**	**Percentage of GFP-ir in mCherry-ir neurons (%)**
99.5 ± 12.1	10.3 ± 3.5	9.55 ± 2.15

*n = 4, 3 sections for each mouse. ir, immunoreactive*.

### Retrograde Tracing of Global Inputs to NPY Neurons in the NAc

**Figure 6:**
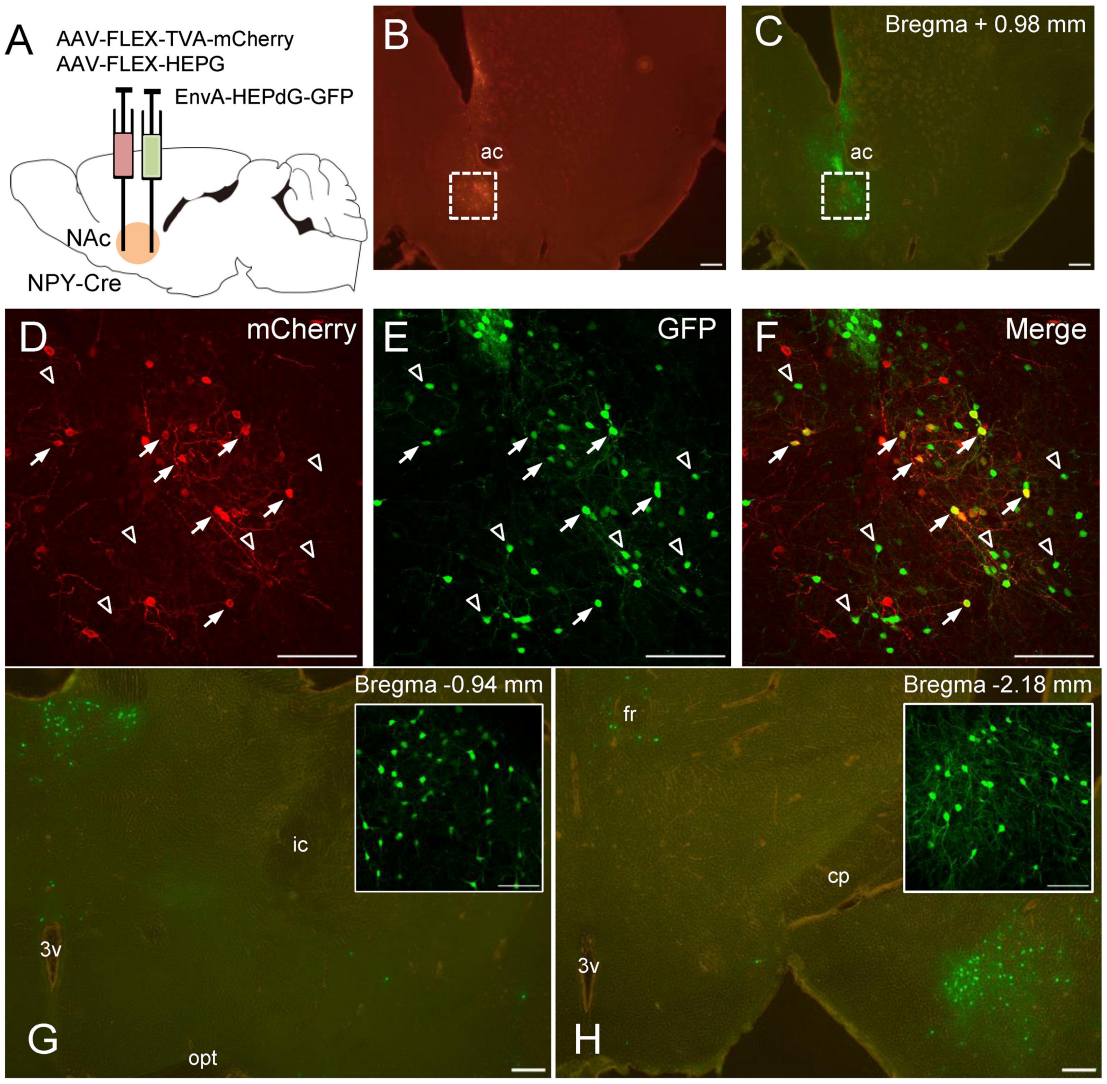
Schematic illustration of AAV(dj)-FLEX-TVA-mCherry, AAV(dj)-FLEX-SADcvsG, and EnvA-HEPdG-GFP injection into the NAc in NPY-Cre mice **(A)**. Representative photographs of AAV(dj)-FLEX-TVA-mCherry **(B)** and EnvA-HEPdG-GFP **(C)** injection sites. Fluorescence image of mCherry **(D)**, GFP **(E)**, and merged **(F**) in the NAc. Arrows show mCherry and GFP double-positive neurons, representing first-infected NPY neurons in the NAc. Open arrowhead shows GFP-positive/mCherry-negative neurons, which are neurons projecting to accumbal NPY neurons. GFP-positive neurons were also found mainly in the midline thalamic nuclei **(G)** and the pBMA **(H)**. Boxed regions in **G** and **H** indicate the midline thalamic nucleus and the pBMA, respectively. Scale bars, 100 or 50 μm (inset). Numerical values in each photomicrographs show distances from bregma (mm). ac, anterior commissure; cp, cerebral peduncle; fr, fasciculus retroflexus; ic, internal capsule; opt, optic tract; 3v, third ventricle.

Finally, we performed global mapping of neurons projecting directly to the NAc NPY neurons by monosynaptic retrograde tracing with rRABV (for coronal and sagittal sections, *n* = 3 each). To this end, we injected AAV(dj)-FLEX-TVA-mCherry and AAV(dj)-FLEX-SADcvsG into the NAc of NPY-Cre mice. TVA and SADcvsG are required for EnvA-HEPdG-GFP infection and subsequent retrograde spread, respectively. After 14 days, EnvA-HEPdG-GFP was injected into the same area, and the brain was analyzed 7 days later (Figure 6A). Successful infection and expression of TVA and EnvA-HEPdG-GFP in the NAc were confirmed by mCherry and GFP immunostaining, respectively (Figures 6B,C). The first-infected cells were identified by coexpression of TVA-mCherry and GFP. In our experiment, neurons expressing both mCherry and GFP were detected only in the NAc (Figures 6D–F). At the injection site, there were some GFP-positive/mCherry-negative neurons, indicating that they were interneurons projecting to the NPY neurons in the same area (Figures 6D–F). Furthermore, we evaluated input neurons that were labeled retrogradely from NPY neurons in the NAc. We found that the vast majority were located in two regions, the midline thalamic nuclei (Figure 6G) and posterior regions of the basomedial amygdala (pBMA; Figure 6H). We also observed a smaller number of GFP-positive neurons in other areas, such as the parafascicular thalamic nucleus, mPFC, entorhinal cortex, insular cortex, prelimbic cortex, posterior region of the basolateral amygdala, lateral preoptic area, and VP.

## Discussion

There are many reports suggesting afferent and efferent connections of neurons in the NAc (Heimer et al., 1991; Brog et al., 1993; Usuda et al., 1998; Itoga et al., 2019). To know the neural connection more deeply, in this study, we focused on NPY neurons in the NAc and sought to clarify the anatomical connections of these neurons using NPY-Cre mice. Our present findings provide novel insight into the projections of NAc NPY neurons to the LH. We performed anterograde and retrograde tracing using AAVs and rRABV and validated these findings with IHC. In addition, we also discovered that the main origins of neurons projecting directly to the NPY neurons in the NAc are the midline thalamic nuclei and pBMA.

Our results from NAc NPY labeling using Cre-dependent AAV and NPY-Cre mice are consistent with previous observations that neurons in the NAc project to the LH in rats (Usuda et al., 1998). We also performed overexpression of synaptophysin fused with EGFP exclusively in the NPY neurons of the NAc. Because synaptophysin is a synaptic vesicle protein and is preferentially expressed in axon terminals, it is often used to label projection terminals (Inoue et al., 2019; Ip et al., 2019). Although NPY-positive cell bodies are very few in the LH, there are many NPY-positive fibers in this area (Chronwall et al., 1985; de Quidt and Emson, 1986). However, the origins of these NPY fibers remain unclear. Our current findings indicate that a subset of NPY fibers in the LH originate from the NAc in mice.

As NPY acts as a neurotransmitter, it is not always localized in the perikaryal region after synthesis, depending on the neuronal activity. In vasopressin neurons, it was reported that their mRNA was expressed, but the peptide was not detected in the cell body in the paraventricular hypothalamic nucleus after salt loading (Amaya et al., 1999). If NPY neurons are projection neurons, retrograde labeling at the cell body level might be hard upon their activity using retrograde tracers, such as CTb, fluorogold, and NPY IHC. In comparison, visualization of NPY neurons using transgenic mice and Cre-dependent AAV seems to detect cell bodies consistently, and it was useful for retrograde labeling of NPY neurons in this study. In addition, we used another retrograde labeling method employing rRABV and found that NPY neurons in the caudal part of the NAc predominantly project to the LH. This result is consistent with a previous anterograde tracing study using BDA showing that neurons in the caudal part of the NAc innervate the LH, although that study did not mention neurochemical type (Usuda et al., 1998).

Five G-protein-coupled receptors for NPY—Y1, Y2, Y4, Y5, and Y6—were found in the rodent brain, and all subtypes are expressed in the LH (Fetissov et al., 2004). Functionally, overexpression of NPY in the LH induces a long-term increase in food intake in rats (Tiesjema et al., 2007). Notably, the stimulatory effect of NPY injection into the LH on food intake is blocked by pretreatment with Y1 and Y5 antagonists (Gumbs et al., 2020), suggesting that the LH is an important brain region involved in the orexigenic effect of NPY *via* Y1 and Y5 receptors. These findings together with our current results suggest that NPY neurons projecting from the NAc to the LH are at least partly involved in feeding behavior. The LH is a major source of orexin and melanin-concentrating hormone (MCH)-expressing neurons (Bittencourt et al., 1992; Sakurai et al., 1998). Previous reports suggested that orexin- and MHC-expressing neurons in the LH receive synaptic inputs from the NAc in mice using nontoxic C-terminal fragments of tetanus toxin and rRABV, respectively (Sakurai et al., 2005; Sanathara et al., 2021). These two neurons innervate a wide range of brain regions and are involved in feeding behavior (Lee et al., 2021). These results indicate that accumbal NPY neurons projecting to the LH may act on orexin and/or MCH neurons to regulate feeding behavior. Further studies are needed to investigate which neurons in the LH receive NPY signaling from the NAc.

Using monosynaptic retrograde tracing with rRABV, we are, to our knowledge, the first to reveal that the afferent inputs to the NPY neurons in the NAc originate mainly from two regions, the midline thalamic nuclei, including the paraventricular thalamic nucleus (PVT), and pBMA. Neuronal connections between the midline thalamic nuclei or pBMA and the NAc are consistent with a previous retrograde study showing that retrograde AAV(retro)-tdTomato injection into the NAc of wild-type mice results in tdTomato expression in the midline thalamic nuclei and the BMA (Itoga et al., 2019). The NAc receives glutamatergic inputs from the PVT, and these inputs modulate hedonic feeding (Christoffel et al., 2021). Thus, NPY neurons in the NAc are directly regulated by excitatory input from PVT neurons and may modulate feeding behavior. Millan et al. reported that the NAc and pBMA mediate cue-triggered alcohol-seeking behavior (Millan et al., 2015). Anterograde tracing using PHA-L revealed that the pBMA mainly innervates the NAc (Petrovich et al., 1996). Thus, the pBMA could modulate reward-seeking behavior *via* NPY neurons in the NAc. Additional studies are needed to address whether neural inputs from the PVT and pBMA regulate the activity of NAc NPY neurons to modulate behaviors, such as feeding and alcohol seeking.

We also found some brain regions innervating NPY neurons in the NAc. A study combining electron microscopy and IHC identified a few synaptic junctions between glutamic acid decarboxylase-labeled terminals and NPY-labeled dendrites in the NAc (Massari et al., 1988). Because there are few GABAergic neurons in the PVT (Ottersen and Storm-Mathisen, 1984), the origins of GABAergic inputs to the NPY neurons in the NAc may be interneurons in the same nucleus or projection neurons from other regions, such as the lateral preoptic area, which contains many GABAergic neurons (Tsukahara and Yamanouchi, 2003). The NAc receives major dopaminergic inputs from the A10 region of the ventral tegmental area (VTA) (Ungerstedt, 1971). In this study, we did not detect GFP-positive cells in the VTA. This result is consistent with a previous study showing few direct synaptic contacts between tyrosine hydroxylase-positive axon terminals and NPY-positive dendrites in the NAc (Aoki and Pickel, 1988), suggesting that NPY neurons in the NAc do not directly connect with dopaminergic neurons from the VTA.

Our study has limitations. Because we used the local injection of AAV to label NPY neurons in the NAc, we did not visualize all accumbal NPY neurons in mice. We only found that 7–10% are projection types to the LH among the accumbal NPY neurons, which are able to visualize by AAV. Additionally, we cannot exclude the possibility that NPY neurons in the anterior part of the bed nucleus of the stria terminalis (BNST) also project to the LH, because the border of the posterior part of the NAc and anterior part of the BNST is ambiguous in mice, and previous reports suggested that NPY-expressing neurons exist in the anterior BNST (Walter et al., 2018) and neurons in the anterior BNST project to the LH (Dong and Swanson, 2004). In this study, we injected a low volume of AAV into the anterior parts of the NAc to minimum leak of AAVs to the anterior BNST. As a result, we confirmed that the spread of AAV was until the region where left and right anterior commissure locate separately, which are shown in Figure 1. Since this region is likely to be the posterior part of the NAc, we considered that the origin of NPY neurons projecting to the LH is the NAc predominantly.

In conclusion, we anatomically characterized NPY neurons in the NAc. NPY neurons were previously considered principally aspiny intrinsic neurons by IHC and electron microscopy (Massari et al., 1988). In this study, we revealed that 7–10% of accumbal NPY neurons are projection neurons. This discrepancy may be caused by differences in the methods for visualizing NPY-positive cell bodies. Previous studies using anti-NPY immunolabeling might have failed to detect the small number of NPY projection neurons in the NAc that send NPY to the axon terminals. It was difficult to detect NPY terminals originating from the NAc in other regions, suggesting that most remaining NPY neurons in the NAc may be interneurons. These findings should provide a foundation for further studies aimed at clarifying the functional importance of NPY projection neurons and NPY interneurons in the NAc.

## Data Availability Statement

The original contributions presented in the study are included in the article, further inquiries can be directed to the corresponding author.

## Ethics Statement

The animal study was reviewed and approved by The Animal Care and Use Committee of the Kyoto Prefectural University of Medicine.

## Author Contributions

SY, NK, and KT performed histological experiments. TM generated the plasmid for AAV and rRABV. AT generated AAV. SY and MT wrote the paper. MT supervised the whole study. All authors contributed to the article and approved the submitted version.

## Funding

This work was supported by the Japan Society for the Promotion of Science Grants-in-Aid [grant no. 17H03553 to MT and grant no. 15K21286 and 19K09032 to SY]

## Conflict of Interest

The authors declare that the research was conducted in the absence of any commercial or financial relationships that could be construed as a potential conflict of interest.

## Publisher's Note

All claims expressed in this article are solely those of the authors and do not necessarily represent those of their affiliated organizations, or those of the publisher, the editors and the reviewers. Any product that may be evaluated in this article, or claim that may be made by its manufacturer, is not guaranteed or endorsed by the publisher.
